# Suppressor of Zeste 12 homolog RNA interference inhibits retinoblastoma cell invasion

**DOI:** 10.3892/ol.2014.2462

**Published:** 2014-08-20

**Authors:** MIN ZHOU, JIANNAN SUN, YUJING LIU, JIA MA

**Affiliations:** Department of Ophthalmology, The First Affiliated Hospital, Beihua University, Jilin City, Jilin 132011, P.R. China

**Keywords:** suppressor of Zeste 12 homolog, RNA interference, retinoblastoma, invasion

## Abstract

Suppressor of Zeste 12 homolog (SUZ12) is known to regulate tumor phenotype through altering gene expression, with an important regulatory role in tumor genesis and development. SUZ12 has been widely investigated; however, no studies regarding the role of the SUZ12 gene in retinoblastoma (RB) have been conducted. In this study, SUZ12 small interfering (si)RNA was transfected into SO-RB50 human RB cells. The influence of SUZ siRNA on RB cell invasion was detected using a soft agar colony forming assay and a Transwell cabin model. The effect of the SUZ12 siRNA on the expression levels of the associated proteins, vascular endothelial growth factor (VEGF), matrix metalloproteinase (MMP)-9 and MMP-2, was detected by western blotting. The number of cell clones was found to be reduced by the siRNA in a dose-dependent manner, and the number of cells that had permeated through the filter membrane was reduced following transfection with the siRNA. SUZ12 inhibition resulted in a marked reduction in VEGF, MMP-2 and MMP-9 expression levels (0.26±0.04, 0.16±0.02 and 0.12±0.02, respectively) compared with the levels in the non-transfected group (0.80±0.10, 0.94±0.16 and 1.15±0.18, respectively) (P<0.01). In conclusion, SUZ12 siRNA inhibited cell invasion and the expression of VEGF, MMP-2 and MMP-9 in SO-RB50 retinoblastoma cells.

## Introduction

Retinoblastoma (RB) is a rare malignant tumor in infants and young children that, if left untreated, has the potential to greatly endanger vision and life (1). Numerous genes have been implicated in the genesis and development of RB (2). Suppressor of Zeste 12 homolog (SUZ12), is an important component of polycomb group protein (PcG), and is essential in cell proliferation, cell cycle and embryonic development processes (3,4). SUZ12 is also known to regulate tumor phenotype through altering gene expression, with an important regulatory role in tumor genesis and development, and this has been widely investigated (5). However, to the best of our knowledge, no studies have analyzed the role of SUZ12 in RB and the underlying mechanism of action. The present study aimed to define the impact of SUZ12 on RB cell invasive ability, along with the potential underlying regulatory mechanism, with the aid of an SUZ12 RNA interference technique in the SO-RB50 RB cell strain.

## Materials and methods

### Experimental materials

The SO-RB50 human RB cell strain was obtained from the Cell Bank of the Chinese Academy of Sciences (Beijing, China) and stored under liquid nitrogen in the laboratory. Dulbecco’s modified Eagle’s medium supplemented with 10% fetal bovine serum was purchased from Gibco-BRL (Carlsbad, CA, USA). The rabbit anti-human SUZ12 (75 kDa), matrix metalloproteinase (MMP)-2 and MMP-9 antibodies, and the vascular endothelial growth factor (VEGF) polyclonal antibodies were purchased from Santa Cruz Biotechnology, Inc. (Santa Cruz, CA, USA). The liposome Oligofectamine (Invitrogen Life Technologies, Carlsbad, CA, USA), SUZ12 small interfering (si)RNA double-stranded oligonucleotides (Invitrogen Life Technologies), Transwell chamber models (Chemicon, Temeluca, CA, USA) and western blotting kits (Boster Bio-company, Beijing, China) were also used in the present study.

### SUZ12 siRNA sequence construction and transfection into the SO-RB50 RB cell strain

The purchased SUZ12 siRNA oligonucleotide sequences used in gene sequencing were as follows: S1, UUA UUG GAC AAC UUA CAU CCU UCC U; S2, AAU UCA UUA CUG GAA ACU GCC AGG G and S3, UAA AUU CUC UUC UUC CUG GAC GAG U. These oligonucleotides were matched with GeneBank human SUZ12 cDNA sequences using Basic Local Alignment Search Tool contrast (http://blast.ncbi.nlm.nih.gov/Blast.cgi). In addition, the following negative control sequence: Sn, UUC UCC GAA CGU GUC ACG UUU GUG C was designed and synthesized. SO-RB50 cells were divided into six groups: Blank control (Con-B), empty vector (Con-N), S1 transfection (S1), S2 transfection (S2), S3 transfection (S3) and Sn transfection (Sn). Each sequence (100 nm) was transfected into the SO-RB50 cells (1×10^5^ cells/ml) using Oligofectamine, with phosphate-buffered saline and empty vector at the same concentrations transferred to the Con-B and Con-N cell groups, respectively. The subsequent procedures did not differ among groups. The most effective siRNA for SUZ12 knockdown was selected for subsequent experiments.

### Western blot analysis of protein expression levels

Exponentially growing SO-RB50 cells were lysed in RIPA buffer and centrifuged at 4,472 × g for 5 min at 4°C. The cell supernatants were collected and the protein levels were determined using the bicinchoninic acid protein quantity detection kit (AR0146; Boster Bio-company) according to the manufacturer’s instructions. Subsequently, 50 μg protein extract was added to 2X sample buffer and denatured at 100°C for 5 min. The proteins were separated by SDS-PAGE and then transferred to nitrocellulose membranes. The membranes were incubated with specific primary antibodies (1:100) at 4°C overnight and mouse anti-rabbit secondary antibodies (1:1,000) (Boster Bio-company) for 4 h and washed with Tris-buffered saline for 5 min. Protein bands were developed with enhanced chemiluminescence (Biosdec Biocompany) and exposed to X-ray films. The captured images underwent grayscale analysis using BandScan software (Glyko, Novato, CA, USA).

### Soft agar assay of cell anchorage-independent growth

A suspension of exponentially growing cells (1×10^3^ cells/ml) was prepared. Soft agar (5%) was mixed with medium at a ratio of 1:9 and added to a plate, which was cooled at room temperature. Subsequently, 1.5 ml cell suspension was added to an equal volume of this plated 0.5% soft agar, and the mixture was agitated and incubated at 37°C with 5% CO_2_ for two weeks. The cell colony formation rate was calculated according to the following formula: Colony formation rate (%) = (number of colonies/number of cells incubated) ×100.

### Ex vivo invasion assay

Cell invasive ability was analyzed using a Transwell chamber model (Chemicon). The cell suspension was adjusted to a concentration of 1×10^5^ cells/ml and 50 μl was placed in the top chamber. After 24 h incubation, the cells that had migrated to the lower chamber were fixed with 10% formalin and stained with Giemsa to quantify the number of transmigrated cells.

### Statistical analysis

Data are expressed as the mean ± standard deviation and were processed using SPSS 16.0 software (SPSS, Inc., Chicago, IL, USA). Comparisons of groups were performed using Student’s t-test and P<0.05 was considered to indicate a statistically significant difference.

## Results

### Efficiency of siRNA-mediated SUZ12 knockdown

**Figure 1:**
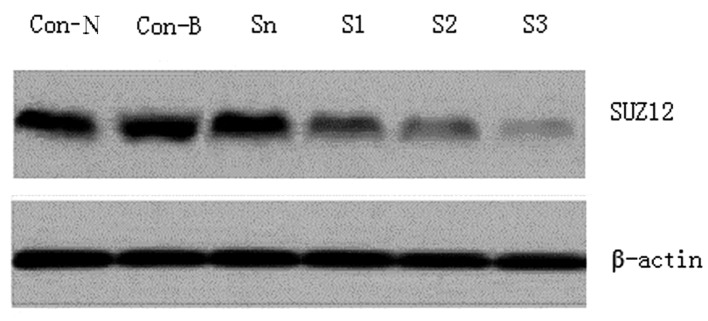
Small interfering (si)RNA-silenced suppressor of Zeste 12 homolog (SUZ12) expression. SUZ12 exhibited high expression levels in the Con-N, Con-B and Sn groups. Following siRNA transfection, marked downregulation of SUZ12 was observed in the S1, S2 and S3 transfection groups, and the reduction was particularly marked in the S3 transfection group.

The SUZ12 expression levels in the SO-RB50 cells in each group were determined using western blotting. SUZ12 exhibited high expression levels in the Con-B, Con-N and Sn groups, but no significant differences were detected among the three groups (P>0.05). By contrast, SUZ12 was significantly downregulated in the SUZ12-siRNA transfection groups (P<0.01); the reduction was particularly marked in the S3 transfection group, which exhibited an ~92.6% decline (Fig. 1).

### Effect of SUZ12 siRNA on anchorage-independent SO-RB50 cell growth

**Figure 2:**
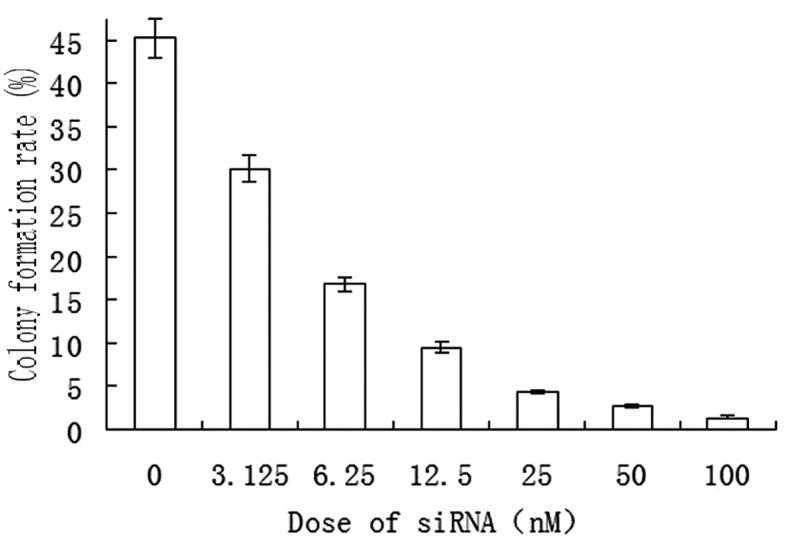
SO-RB50 human retinoblastoma cell colony formation rate was gradually reduced as the suppressor of Zeste 12 homolog small interfering (si)RNA transfection dose was increased.

Due to the above finding that the S3 sequence was the most effective in silencing SUZ12, this sequence was selected as the SUZ12-specific interference sequence in subsequent experiments. A soft agar colony formation assay revealed that SO-RB50 cells formed colonies spontaneously in the *in vitro* culture system. Following transfection of the cells with S3 siRNA (at doses of 0, 3.125, 6.25, 12.5, 25, 50 and 100 nM), a gradually reduced colony formation rate was observed as the transfection dose was increased (Fig. 2).

### Effect of SUZ12 interference on SO-RB50 cell invasion

**Figure 3:**
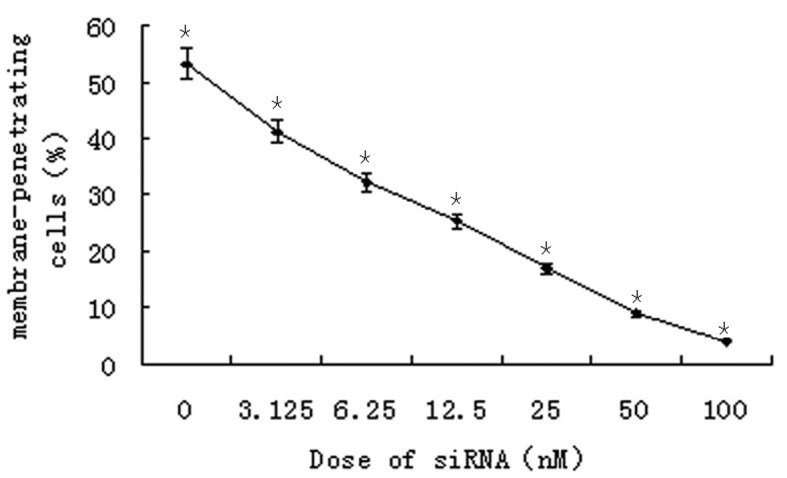
Suppressor of Zeste 12 homolog small interfering (si)RNA significantly reduced the percentage of membrane-permeating SO-RB50 human retinoblastoma cells in a concentration-dependent manner (^*^P=0.018).

A Transwell chamber assay was employed to detect the invasive ability of SO-RB50 cells 48 h after transfection with different concentrations of the S3 siRNA. The results revealed that siRNA-mediated knockdown of SUZ12 significantly reduced the number of membrane-permeating cells in a concentration-dependent manner (P=0.018; Fig. 3).

### Effect of SUZ12 interference on VEGF, MMP-2 and MMP-9 protein expression levels

**Figure 4:**
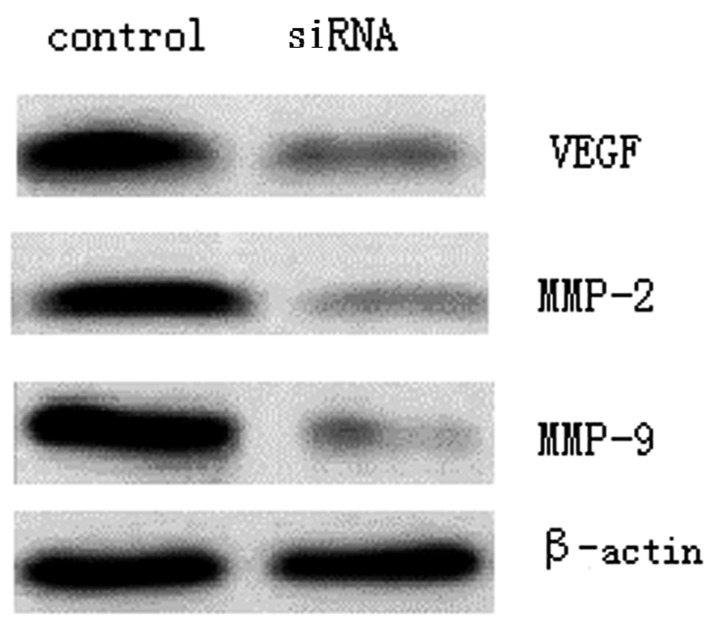
Suppressor of Zeste 12 homolog small interfering (si)RNA downregulated the expression of vascular endothelial growth factor (VEGF), matrix metalloproteinase (MMP)-2 and MMP-9 proteins.

To identify the underlying mechanism of SUZ12-mediated regulation of RB cell invasion, the VEGF, MMP-2 and MMP-9 expression level changes following SUZ12 interference were detected using western blotting, whereby the grey values represented the level of protein expression. The results revealed that the VEGF level (0.26±0.04) was significantly reduced compared with that prior to SUZ12 knockdown (0.89±0.10) (P<0.01). Furthermore, the levels of MMP-2 (0.16±0.02) and MMP-9 (0.12±0.02) were also lowered significantly from those prior to SUZ12 knockdown (0.94±0.16 and 1.15±0.18, respectively) (P<0.01; Fig. 4).

## Discussion

SUZ12 is a component of the PcG complex that, along with zeste 2 enhancer and embryonic ectoderm development, is involved in cell proliferation, differentiation and aging via inactivating target gene promoters (6). SUZ12 is critical for tumor pathogenesis and development (7), and may be involved in the regulation of tumor stem cells (8). Previous studies demonstrated high SUZ12 expression levels in aggressive tumors, such as prostatic carcinoma (9), breast carcinoma (10) and nervous system carcinoma (11), and a marked correlation between SUZ12 expression levels and tumor malignancy. However, to the best of our knowledge, no studies have analyzed the role of SUZ12 in RB and the underlying mechanism of action.

In the present study, siRNA-mediated knockdown of SUZ12 was performed, and the cell anchorage independence and invasive ability were observed using soft agar colony formation assay and Transwell chamber models, respectively. Anchorage dependence refers to the finding that certain cells require anchorage with a specific substrate to suppress apoptosis and survive; conversely, tumor cells are characterized by anchorage-independent growth. The soft agar colony formation assay is able to measure tumor cell anchorage-independent growth and tumor malignancy (12). Greater invasive ability in tumor cells is associated with a higher number of cell colonies. The present study revealed that SUZ12-specific siRNA suppressed SO-RB50 cell colony formation in soft agar in a concentration-dependent manner (Fig. 2), demonstrating that SUZ12 interference hindered SO-RB50 cell invasion. The ability of tumors to migrate and invade is associated with the microenvironment and the extracellular matrix (ECM); therefore, a Transwell chamber model that imitates the ECM is thus far a reliable method for assaying cell invasive ability (13). In the present study, a marked reduction in the number of SO-RB50 cells that had passed through the Transwell chamber was detected following SUZ12 silencing, and the reduction appeared to be siRNA concentration-dependent (Fig. 3). Preliminarily, these findings suggest that interference with SUZ12 suppresses RB cell invasive ability.

The invasive and migratory abilities of tumor cells are closely associated with the capacity of the cells to induce proteinase production that may degrade the ECM and the basement membranes (14). A substantial number of molecules are involved in the regulation of tumor cell invasion and migration. Among these, VEGF, which mediates tumor vascularizaion, is important in tumor formation, invasion and metastasis, and may be a promising target in tumor therapy (15). The present study demonstrated that SUZ12 interference suppressed SO-RB50 cell invasion as well as reducing VEGF activity. Another type of molecule that is associated with tumor invasion is the MMPs. Among these, MMP-9 and MMP-2 regulate vascular endothelial cell activity, induce neovascularization, and exert an important role in RB hyperplasia and differentiation (16). Furthermore, upregulation of MMP-9 and MMP-2 is associated with poorer outcomes in RB (17). The present study revealed that SUZ12 interference inhibited MMP-2 and MMP-9 activity, and thus hindered cell invasion.

In conclusion, in the present study, SUZ12 knockdown attenuated the invasive ability of RB SO-RB50 cells and suppressed VEGF, MMP-2 and MMP-9 expression. Therefore, SUZ12 is of great importance in regulating RB invasion and metastasis, and is expected to be involved in targeted molecular therapy in RB.
